# Negative Regulation of Humoral Immunity Due to Interplay between the SLAMF1, SLAMF5, and SLAMF6 Receptors

**DOI:** 10.3389/fimmu.2015.00158

**Published:** 2015-04-14

**Authors:** Ninghai Wang, Peter J. Halibozek, Burcu Yigit, Hui Zhao, Michael S. O’Keeffe, Peter Sage, Arlene Sharpe, Cox Terhorst

**Affiliations:** ^1^Division of Immunology, Beth Israel Deaconess Medical Center, Harvard Medical School, Boston, MA, USA; ^2^Department of Microbiology and Immunology, Harvard Medical School, Boston, MA, USA

**Keywords:** SLAM family receptors, SLAM-associated protein, follicular T helper cells, germinal center B cells, marginal zone B cells, anti-SLAMF6 mAb

## Abstract

Whereas the SLAMF-associated protein (SAP) is involved in differentiation of T follicular helper (Tfh) cells and antibody responses, the precise requirements of SLAMF receptors in humoral immune responses are incompletely understood. By analyzing mice with targeted disruptions of the *Slamf1*, *Slamf5*, and *Slamf6* genes, we found that both T-dependent and T-independent antibody responses were twofold higher compared to those in single knockout mice. These data suggest a suppressive synergy of SLAMF1, SLAMF5, and SLAMF6 in humoral immunity, which contrasts the decreased antibody responses resulting from a defective GC reaction in the absence of the adapter SAP. In adoptive co-transfer assays, both *[Slamf1* + *5* + *6]*^−/−^ B and T cells were capable of inducing enhanced antibody responses, but more pronounced enhancement was observed after adoptive transfer of *[Slamf1* + *5* + *6]*^−/−^ B cells compared to that of *[Slamf1* + *5* + *6]*^−/−^ T cells. In support of *[Slamf1* + *5* + *6]*^−/−^ B cell intrinsic activity, *[Slamf1* + *5* + *6]*^−/−^ mice also mounted significantly higher antibody responses to T-independent type 2 antigen. Furthermore, treatment of mice with anti-SLAMF6 monoclonal antibody results in severe inhibition of the development of Tfh cells and GC B cells, confirming a suppressive effect of SLAMF6. Taken together, these results establish SLAMF1, SLAMF5, and SLAMF6 as important negative regulators of humoral immune response, consistent with the notion that SLAM family receptors have dual functions in immune responses.

## Introduction

The humoral immune response is crucial for protecting individuals from many infections and eliminating foreign substances. Antibody responses can be induced in a T cell dependent or T cell independent manner. In T-independent immunity, the antibody response occurs directly after B cell activation in T cell deficient mice. In contrast with T-independent responses, T-dependent responses require T cell help for B cell activation and maturation. In the presence of T cell help, B cells undergo robust proliferation and somatic hypermutation of their variable region genes and differentiate into high affinity memory B cells and long-lived plasma cells in the germinal centers (1, 2). Although several subsets of CD4^+^ T helper cells may be implicated in T-dependent humoral responses, it becomes clear that the follicular helper CD4^+^ T cell subset [T follicular helper (Tfh)] is a major B cell help provider (3–6). Tfh cells exhibit a phenotype distinct from that of other effector CD4^+^ T helper cells, as they express the transcription factor B cell lymphoma 6 (Bcl6) that is necessary for the development of Tfh cells and inhibits expression of genes critical for development of other T helper cells (4, 7–9). Although Bcl6 expression by pre-Tfh cells is required, it is not sufficient *in vivo* for full polarization of Tfh cells. In fact, multiple molecules have been shown to be involved in the differentiation of Tfh cells (3, 4, 6). In addition, Tfh development is highly dependent on B cell responses, as Tfh cells are not found in B cell deficient mice (7, 10, 11). These findings indicate that, through their interaction, GC B cells and Tfh cells reciprocally provide each other with signaling for survival, proliferation, and differentiation.

The signaling lymphocytic activation molecule family (SLAMF) includes nine structurally related Ig-like proteins that are differentially expressed on the surface of hematopoietic cells (12). SLAMF receptors have been shown to function as co-stimulatory molecules and to modulate the activation and differentiation of a wide array of immune cell types involved in both innate and adaptive immune responses (12–14). While most SLAMF receptors serve as self-ligands, SLAMF2 and SLAMF4 interact with each other. Six SLAMF receptors (SLAMF1, SLAMF3, SLAMF4, SLAMF5, SLAMF6, and SLAMF7) carry one or more copies of an immunoreceptor tyrosine-based switch motif (ITSM) in their cytoplasmic tails. This signaling switch motif can recruit SH2 domain-containing signaling molecules such as SLAM-associated protein (SAP) (15). SAP is a cytoplasmic adapter molecule with a single Src homology 2 domain and a small carboxy-terminal region. The SAP family consists of three members: SAP expressing T, NK, and NKT cells, and EAT-2A and EAT-2B (murine) expressing NK cells and APC (12, 16). There is accumulating evidence that SAP and EAT-2 can function as signaling adaptors that link SLAMF receptors to active signaling molecules such as the Src family protein tyrosine kinases Fyn and PI3K (15, 17–21). SAP and EAT-2 have also been shown to act as blockers to outcompete SH2 domain-containing inhibitory molecules SHP1, SHP2, and SHIP1 (22–28).

Deficiencies in the gene that encodes SAP (*SH2D1A*) result in a primary immunodeficiency called X-linked lymphoproliferative disease (XLP) (29–31). Patients with XLP suffer from fatal infectious mononucleosis, malignant B cell lymphomas, and dysgammaglobulinemia. Defects in humoral responses and lack of germinal center formation are observed in XLP patients and in virally infected or immunized SAP-deficient mice (32–36). Considerable evidence indicates that the humoral immune response defect in XLP patients and *SAP*^−/−^ mice stems from a defect in CD4^+^ helper cells because T-dependent antigen responses are defective and are restored after reconstitution with WT CD4^+^ T cells, but not WT B cells (32, 34, 35). However, the role of SAP in T-dependent humoral responses remains unclear. The SLAM/SAP/FynT axis regulates IL-4 producing Th2 differentiation, as demonstrated by the observation that *Slamf1*^−/−^ and *SAP*^−/−^ mice have defective Th2 cytokine secretions (37–39). IL-4 is known to stimulate B cell antibody responses and Ig class switching, but the R78A mutant SAP mice can mount normal T-dependent antibody responses even though this mutant SAP molecule loses Fyn binding motif R78 (40). Recently, Qi and co-workers elegantly showed that SAP-deficient CD4^+^ T cells cannot form lasting mobile conjugate pairs with cognate B cells in the germinal center while the interaction between SAP-deficient T cells and DC is not affected (41). Since a sustained T-B conjugate allows their comprehensive activation and subsequent differentiation to Tfh cells and GC B cells, unstable T-B cell conjugates may contribute to humoral immune deficiency in *SAP*-deficient mice and XLP patients.

Compared to severe immunodeficiencies in *SAP*^−/−^ mice, single ablation of SLAMF receptors causes a mild phenotype (40, 42–44). When mice deficient in SLAMF1 (the prototypic member of the SLAMF receptors) were infected with LCMV, Tfh cell differentiation, germinal center development, and the acute or long-term anti-viral antibody responses were comparable between LCMV-infected WT and *Slamf1*^−/−^ mice (40). Similar to *Slamf1*^−/−^ mice, *Slamf3*^−/−^*, Slamf5*^−/−^, and *Slamf6*^−/−^ mice showed no defects in response to LCMV (28, 42). This suggests that functional redundancy exists among the SLAMF receptors, which has been confirmed in NKT cell development in pseudo *Slamf[1* + *6]-*deficient bone marrow reconstituting mice (45) and recent *Slamf[1* + *6]*^−/−^ mice (46). For more than a decade, investigating the role of SLAMF receptors in SAP-mediated signaling has been difficult due to an inability to generate double or multiple SLAMF receptor deficient mice, as the receptors are closely located on the same chromosome (12). To define roles of SLAMF receptors as well as how they interact with one another in humoral immune responses, we generated *Slamf[1* + *6]* double knockout and *Slamf[1* + *5* + *6]* triple knockout mice using a two-time gene targeting technique and Cre/LoxP system. Surprisingly, we found that the combined absence of SLAMF1, SLAMF5, and SLAMF6 results in higher antibody production in response to both T-dependent and T-independent antigens. In addition, the administration of anti-SLAMF6 monoclonal antibody also impairs humoral immune responses *in vivo*. These observations suggest that SLAMF1, SLAMF5, and SLAMF6 function as negative regulators in T-dependent and T-independent antibody responses.

## Materials and Methods

### Mice

**Figure 1:**
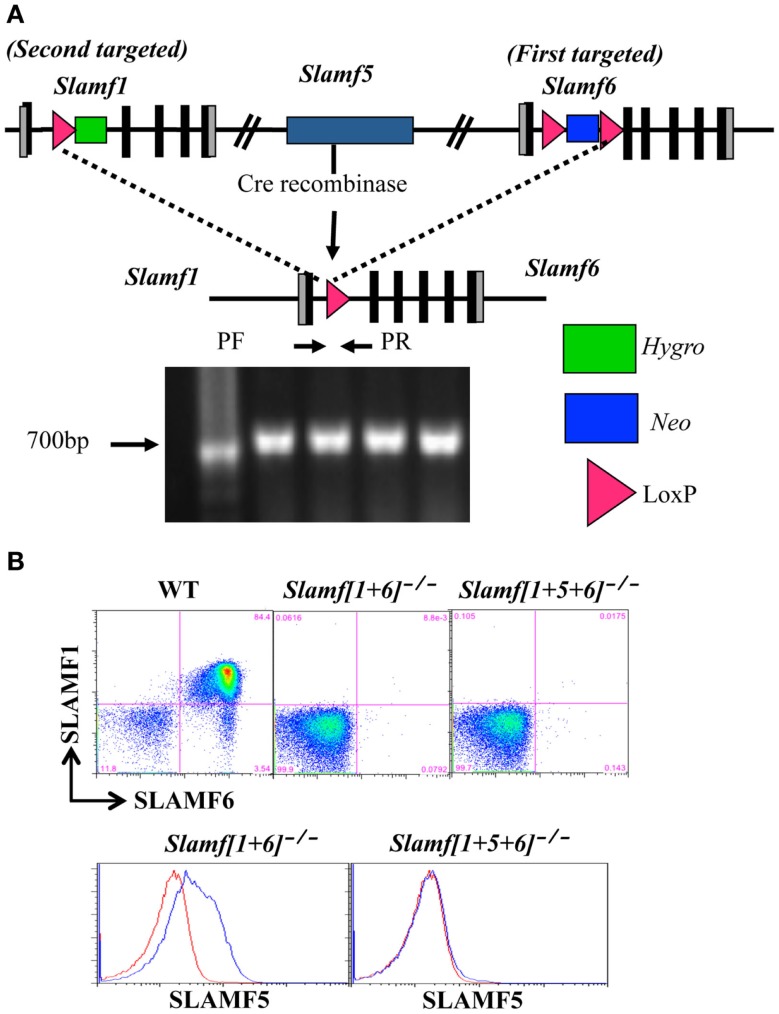
**Generation of *Slamf[1* + *6]*^−/−^ and *Slamf[1* + *5* + *6]*^−/−^ mice**. **(A)** Schematic representation of the *Slamf[1* + *6]* and *Slamf[1* + *5* + *6]* targeting strategy. Top: illustration of the genomic mouse SLAMF1-5-6 locus after targeted replacement of exon 2 and 3 of both *Slamf1* and *Slamf6* genes. Middle: The *Slamf*1-5-6 locus after Cre-mediated recombination leading to the deletion of the LoxP site-flanked genomic fragment. Bottom: PCR genotyping primers (PF and PR) used to confirm the junction of Cre-mediated deletion from mouse-tail DNA. **(B)** Thymocytes from WT, *Slamf[1* + *5]*^−/−^, and *Slamf[1* + *5* + *6]*^−/−^ mice were stained with anti-SLAMF1, anti-SLAMF5, and anti-SLAMF6 antibodies, and the expression of SLAMF1, SLAMF5, and SLAMF6 was evaluated by flow cytometry.

To generate *Slamf[1* + *6]*^−/−^ and *Slamf[1* + *5* + *6]*^−/−^ mice, a *B6* bacterial artificial chromosome clone (B6 BAC clone #RP23-77A8) containing the *Slamf1* and *Slamf6* genes was used to construct a targeting vector with a neomycin resistant cassette flanked by two LoxP sites. SLAMF6 ES cell clones heterozygous for the mutation were generated by standard methods. To generate *Slamf1* and *Slamf6* double-deficient mice, we used a SLAMF1 targeting vector to retarget the previously generated SLAMF6 mutant ES cell clone that was known to give germline transmission with extremely high frequency. Co-integration of the two targeting vectors on the same chromosome was assessed by *in vitro* transfection-targeted ES cell clones with a Cre recombinase expression vector. Deletion of the whole *Slamf1*, *Slamf5*, and *Slamf6* locus was confirmed by PCR (Figures 1A,B). B6 background *Slamf5*^−/−^ mice have been reported previously (46). Wild-type C57BL/6 (B6) mice were obtained from the Jackson Laboratory. Animal studies were conducted in accordance with the National Research Council’s Guide for the Care and Use of Laboratory Animals and were approved by the Beth Israel Deaconess Medical Center Institutional Animal Care and Use Committee.

### Anti-SLAMF receptor antibodies

Rat anti-mouse SLAMF1 mAb (9D1) is specific for the extracellular region of mouse SLAMF1 (25). The anti-mouse SLAMF5 mAb was generated by fusing NS1 myeloma cells with spleen cells obtained from Armenian hamsters, which were immunized three times with mouse SLAMF5-Fc fusion protein (47). Mouse anti-mouse SLAMF6 mAbs (13G3 and 330) are specific for the extracellular region of mouse SLAMF6. Anti-SLAMF1, anti-SLAMF5, and anti-SLAMF6 mAbs used in our studies were purified by affinity chromatography (Harlan Bioproducts for Science). Anti-SLAMF6 F(ab’)_2_ fragments were generated from whole SLAMF6 mAb, using the Thermo Scientific Pierce F(ab’)_2_ Preparation Kit (PI-44988) according to manufacturer instructions.

### Immunization

Mice were immunized intraperitoneally (i.p.) with 40 μg of NP-ovalbumin (NP-OVA, Biosearch Technology) precipitated with Complete Freund’s Adjuvant (Difco) or Alum. For eliciting TI antigen responses, mice were i.p. immunized with 20 μg of NP28-Ficoll (Biosearch Technologies) or with 10 μg NP-LPS. Mice were bled on day 9 (for T-dependent antigen) or day 7 (for T-independent antigen) post-immunization. NP-specific IgG and IgM titers were determined by ELISA after serial dilutions of the serum.

### Adoptive cell transfer

Naïve CD4^+^ and B220^+^ B cells were purified from the spleens of WT and *Slamf[1* + *5* + *6]*^−/−^ mice using a magnetic cell sorting kit (Miltenyi Biotec). *Rag-1*^−/−^ recipient mice were injected with 5 × 10^6^ CD4^+^ T cells and 10 × 10^6^ B220^+^ B cells in 200 μl PBS. Mice were immunized with NP-OVA/CFA 7 days after adoptive cell transfer.

### ELISA

Serum was collected from mice on day 7 or 9. High binding plates (Costar) were coated overnight at 4°C with [NP(4)-BSA] or [NP(25)-BSA] (50 μg/ml, Biosearch Technologies). Horseradish peroxidase-conjugated sheep anti-mouse IgG antibody (Amersham) was used for detection. Relative affinity of the NP-specific IgG antibodies was calculated from the ratio of antibody binding to low-density hapten [NP(4)-BSA] versus high-density hapten [NP(25)-BSA] coated plates.

### Flow cytometry

Single-cell suspensions of splenocytes, thymocytes, and inguinal lymph nodes were stained with the following antibodies and reagents after blocking non-specific binding with CD16/32 and 15% rabbit-serum: αCD4 (RM4-5), αPD-1 (RMP1-30), αCD44 (IM7), αCD138 (281-2), αB220 (RA3-6B2), αFas (Jo2), αT- and B-cell activation antigen (GL-7), and αIgD (11-26) purchased from eBioscience, BD Pharmingen, or Biolegend. NP32-phycoerythrin was purchased from Biosearch Technologies (N-5070-1). For staining of CXCR5, biotinylated-αCXCR5 (2G8, BD Biosciences) was used, followed by PE-labeled streptavidin (eBioscience). For staining of NP-PE, splenocytes were fixed for 10 min with 4% paraformaldehyde, then were washed with flow cytometry buffer with 0.2% saponin, and stained in the presence of 0.2% saponin. Data were acquired using an LSRII flow cytometer (BD Pharmingen) and analyzed using FlowJo software, Version 8.8.6 (TreeStar Inc.).

### Statistical analysis

Statistical significance was determined by unpaired *t*-test (two-tailed with equal SD) using Prism software (GraphPad, San Diego, CA, USA). The *p*-value <0.05 was considered statistically significant.

## Results

### Generation of *Slamf[1* + *6]*^−/−^ and *Slamf[1* + *5* + *6]*^−/−^ mice

Since the murine SLAMF1, SLAMF5, and SLAMF6 genes are closely linked on mouse chromosome 1, a mouse strain lacking *Slamf[1* + *6]* or *Slamf[1* + *5* + *6]* cannot be generated by interbreeding individual *Slamf1*^−/−^, *Slamf5*^−/−^, and *Slamf6*^−/−^ mice. To generate *Slamf[1* + *6]*^−/−^ and *Slamf[1* + *5* + *6]*^−/−^ mice, we first replaced exons 2 and 3 of the *Slamf6* gene with a LoxP-flanked PGK-Neo^R^ cassette in the first targeting event in B6 ES cells (Figure 1A). We next transfected one of the SLAMF6-targeted ES cell clones with a vector that replaced exons 2 and 3 of the *Slamf1* gene with a hygromycin resistant gene containing a LoxP site, thus generating *Slamf[1* + *6]*^−/+^ ES cells. To identify ES cell clones in which both insertions had taken place on the same chromosome, we removed the LoxP-flanked chromosome fragment of 200 Kb, which includes the *Slamf1*, *Slamf5*, and *Slamf6* genes. The confirmed *Slamf[1* + *6]*^−/+^ ES cell clones were used to generate *Slamf[1* + *6]*^−/−^ mice. Subsequently, *Slamf[1* + *6]*^−/−^ mice were bred to CreTg mice to obtain *Slamf[1* + *5* + *6]*^−/−^ mice (Figure 1A). The absence of *Slamf[1* + *6]* and *Slamf[1* + *5* + *6]* expression was confirmed by flow cytometric analyses using SLAMF1, SLAMF5, and SLAMF6 specific antibodies (Figure 1B).

### The number of marginal zone B cells is significantly increased in *Slamf[1* + *5* + *6]*^−/−^ mice

**Figure 2:**
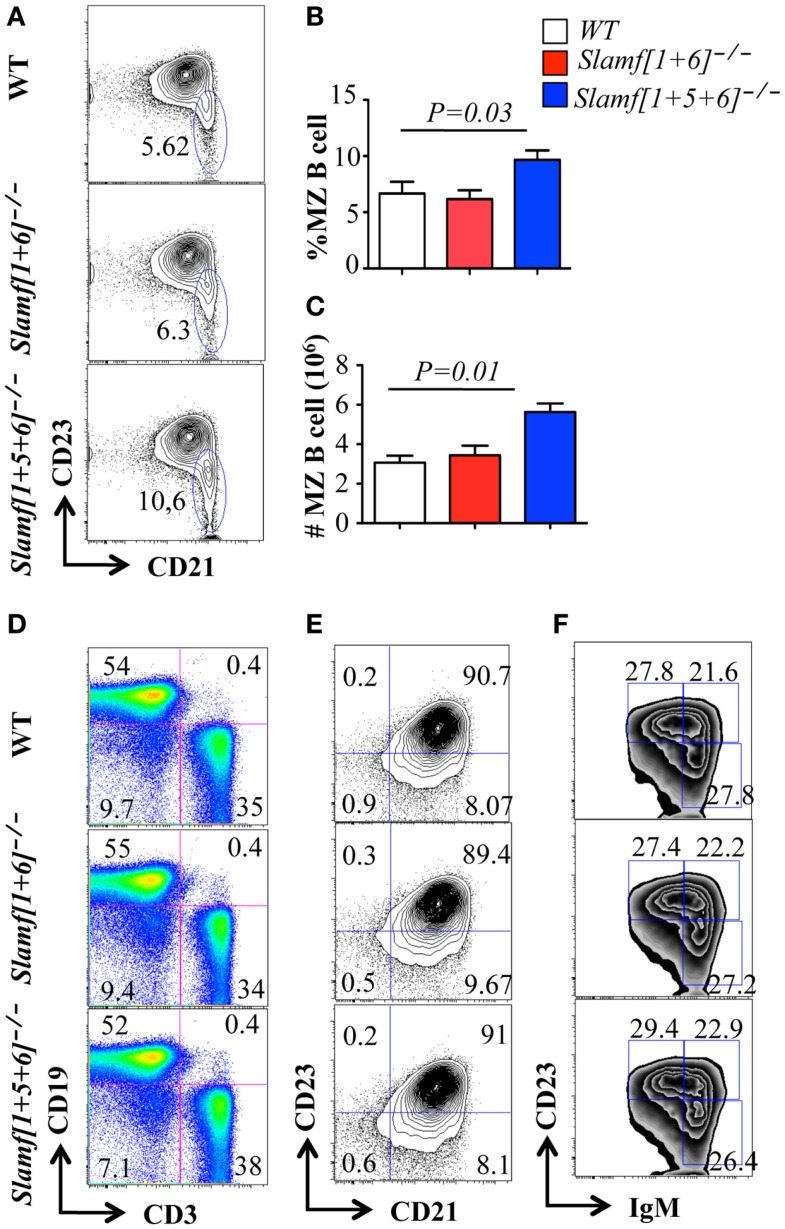
**Comparison of B cell and T cell populations in *Slamf[1* + *5]*^−/−^, *Slamf[1* + *5* + *6]*^−/−^, and WT mice**. Flow cytometric analyses of B cell and T cell subsets in the spleens of *Slamf[1* + *6]*^−/−^, *Slamf[1* + *5* + *6]*^−/−^, and WT mice: **(A)** CD19^+^AA4-IgM^hi^ cells in spleens are gated for the expression of CD23 and CD21 to delineate CD21^+^CD23^-^ marginal zone (MZ) B cells. **(B)** Percentage of CD19^+^AA4^−^ IgM^hi^CD21^+^ CD23^−^ MZ B cells. **(C)** The number of CD19^+^AA4^−^ IgM^hi^CD21^+^CD23^-^ MZ B cells. **(D)** Splenocytes from *Slamf[1* + *6]*^−/−^, *Slamf[1* + *5* + *6]*^−/−^, and WT mice are stained for surface expression of CD3 and CD19. **(E)** CD19^+^ AA4^−^ IgM^hi^CD21 cells in spleens are gated for the expression of CD23 and CD21 to delineate CD21^+^CD23^+^ follicular B cells in the spleens. **(F)** Transitional B cell subsets in spleens are stained for the expression of CD19, AA4.1, CD23, and IgM: T1 (CD19^+^AA4^+^IgM^hi^CD23^-^), T2 (CD19^+^AA4^+^IgM^hi^CD23^+^), and T3 (CD19^+^AA4^+^IgM^lo^CD23^-^).

Thymic development and the number of splenic T cells were not altered in *Slamf[1* + *6]*^−/−^ or *Slamf[1* + *5* + *6]*^−/−^ mice (Figures S1A,C in Supplementary Material). However, a close examination of the B cell compartment by flow cytometric analysis revealed that the number and percentage of marginal zone (MZ) B cells (sIgM^hi^AA4.1^-^CD19^+^CD21^hi^CD23^lo-neg^) was significantly increased in *Slamf[1* + *5* + *6]*^−/−^ mice as compared to WT and *Slamf[1* + *6]*^−/−^ mice (Figures 2A–C). By contrast, the percentage and number of T and B cells and the number of splenocytes in *Slamf[1* + *5* + *6]*^−/−^ mice were identical to those in WT mice (Figure 2D; Figures S1A–C in Supplementary Material). The population of follicular B cells (AA4.1^−^ CD21^+^CD23^+^sIgM^int^) in the spleens of *Slamf[1* + *6]*^−/−^ and *Slamf[1* + *5* + *6]*^−/−^ mice was similar to that in WT mice (Figure 2E). Similarly, the frequencies of CD5^+^ B1a and CD5^−^ B1b cells were comparable in the peritoneal cavity of mutant and WT mice (Data not shown). Immature peripheral B cells can be divided into three transitional populations based on their surface marker expression, designated as transitional type 1 (T1) (AA4.1^+^sIgM^int^CD23^-^), transitional type 2 (T2) (AA4.1^+^sIgM^hi^CD23^-^), and transitional type 3 (T3) (AA4.1^+^sIgM^int^CD23^+^). Similarly, the frequencies of T1, T2, and T3 immature B cell populations in *Slamf[1* + *6]*^−/−^ and *Slamf[1* + *5* + *6]*^−/−^ mice were not significantly different from those of wild-type mice (Figure 2F). Collectively, these findings show that the ablation of SLAMF1, SLAMF5, and SLAMF6 affects the development of MZ B cells, but other B cell subsets and T cell development occur normally in *Slamf[1* + *6]*^−/−^ and *Slamf[1* + *5* + *6]*^−/−^ mice.

### Enhanced T cell dependent antibody production in *Slamf[1* + *5* + *6]*^−/−^ mice

**Figure 3:**
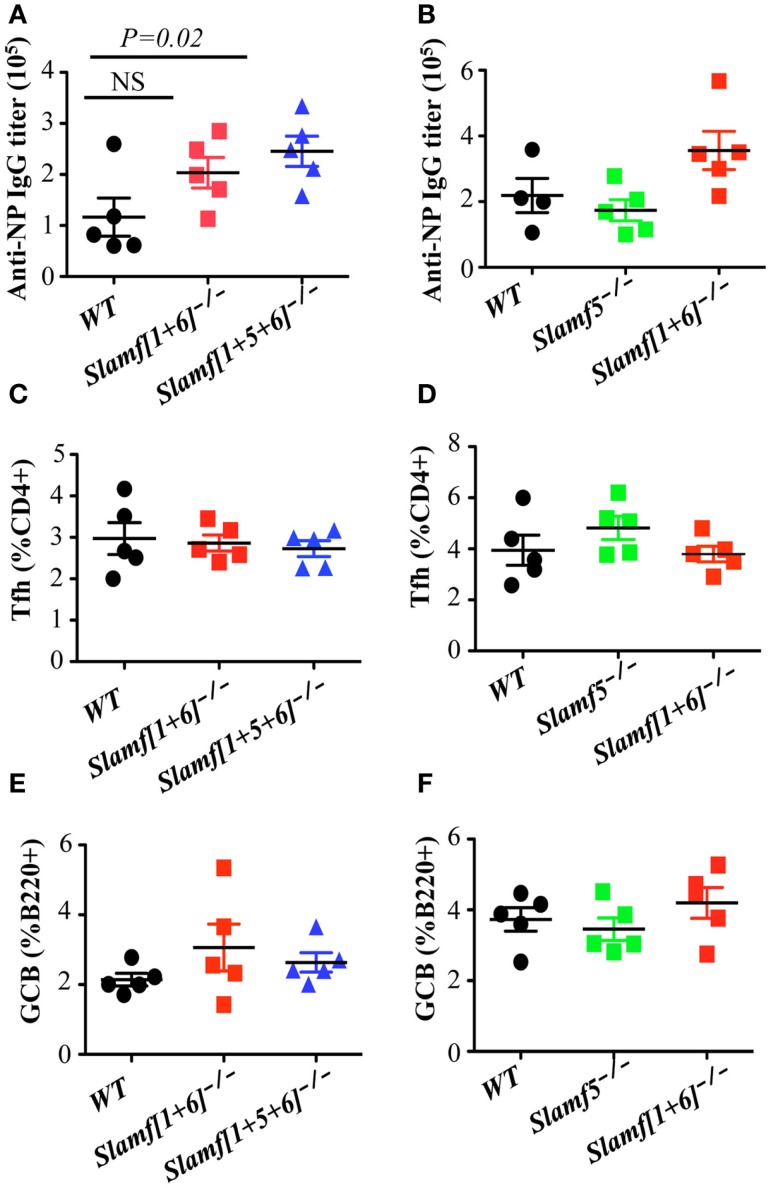
**A combination of SLAMF1, SLAMF5, and SLAMF6 negatively regulates T cell dependent antibody responses, but normal Tfh and GCB development is observed in *Slamf[1* + *5* + *6]*^−/−^ mice**. WT, *Slamf5*^−/−^, *Slamf[1* + *6]*^−/−^, and *Slamf[1* + *5* + *6]*^−/−^ mice were immunized with 40 μg of NP-OVA and serum was collected on day 9. **(A)** NP-specific IgG titers for *Slamf[1* + *6]*^−/−^*, Slamf[1* + *5* + *6]*^−/−^, and WT mice immunized with NP-OVA in CFA were determined by ELISA using NP(4)-BSA coated plates. **(B)** NP-specific IgG titers for *Slamf5*^−/−^*, Slamf[1* + *6]*^−/−^, and WT mice immunized with NP-OVA in Alum were determined by ELISA using NP(4)-BSA coated plates. **(C)** Percentage of Tfh cells (CD4^+^PD-1^+^CXCR5^+^) in the spleens of *Slamf[1* + *6]*^−/−^*, Slamf[1* + *5* + *6]*^−/−^, and WT mice. **(D)** Percentage of Tfh cells (CD4^+^PD-1^+^CXCR5^+^) in the spleens of *Slamf5*^−/−^*, Slamf[1* + *6]*^−/−^, and WT mice. **(E)** Percentage of Germinal Center B cells (B220^+^GL7^+^Fas^+^) in the spleens of *Slamf[1* + *6]*^−/−^*, Slamf[1* + *5* + *6]*^−/−^, and WT mice. **(F)** Percentage of Germinal Center B cells (B220^+^GL7^+^Fas^+^) in the spleens of *Slamf5*^−/−^*, Slamf[1* + *6]*^−/−^, and WT mice. Data represent at least three independent experiments.

Although most SLAMF receptors are expressed on the surface of T and B cells, ablation of single SLAMF genes does not lead to significant defects in germinal center formation after protein immunization or viral infection (27, 42, 44). In contrast, the absence of SAP, the SLAMF specific adaptor, leads to a severe defect in humoral response (14, 32, 34), which suggests functional redundancies in the control of antibody responses by SLAMF receptors. To test this hypothesis, we compared NP-specific antibody production by *Slamf[1* + *6]*^−/−^, *Slamf[1* + *5* + *6]*^−/−^, and WT mice. *Slamf[1* + *6]*^−/−^ mice, which had been immunized with NP-OVA in CFA, produced similar amounts of NP-specific serum IgM as WT mice (data not shown). However, the level of anti-NP IgG in the serum of *Slamf[1* + *6]*^−/−^ mice was consistently higher, although statistical analysis did not reach significance (Figure 3A). Surprisingly, the additional disruption of the *Slamf5* gene significantly augmented the level of anti-NP IgG in *Slamf[1* + *5* + *6]*^−/−^ mice (Figure 3A) even though NP-specific IgM production was not altered (data not shown). Affinity maturation of NP-specific IgG was comparable between the mutant and WT mice (data not shown). Because *Slamf[1* + *5* + *6]*^−/−^ mice produced higher NP-specific IgG compared to *Slamf[1* + *6]*^−/−^ mice, we reasoned that SLAMF5 signaling might suppress antibody responses. To test this, we then immunized *Slamf5*^−/−^ mice with NP-OVA. In contrast to a previous report (41), *Slamf5* deficiency had no effect on NP-specific antibody production or the development of Tfh cells or GC B cells (Figures 3B–F). Taken together, the data support the notion that SLAMF1, SLAMF5, and SLAMF6 cooperate in the negative regulation of T-dependent antibody responses.

### The combined absence of SLAMF1, SLAMF5, and SLAMF6 enhances antigen specific plasma cell expansion, but has no effect on the development of GC B cells, Tfh cells, or T follicular regulatory (Tfr) cells

As strong humoral immune responses, characterized by GC formation and long-lived plasma and memory B cells, are dependent on help provided by CD4^+^ Tfh cells (4, 5, 48), we next examined whether enhanced T-cell dependent antibody responses in *Slamf[1* + *5* + *6]*^−/−^ mice are correlated with an increase in Tfh cell differentiation and higher germinal center responses after immunization with NP-OVA. Contrary to our prediction, the percentage and number of Tfh cells was comparable between *Slamf[1* + *5* + *6]*^−/−^ and WT mice (Figure 3C; Figure S2A in Supplementary Material). Percentages of GC B cells (FAS^+^ GL-7^+^) were also unaffected by the combined absence of SLAMF1, SLAMF5, and SLAMF6 (Figure 3E; Figure S2B in Supplementary Material).

**Figure 4:**
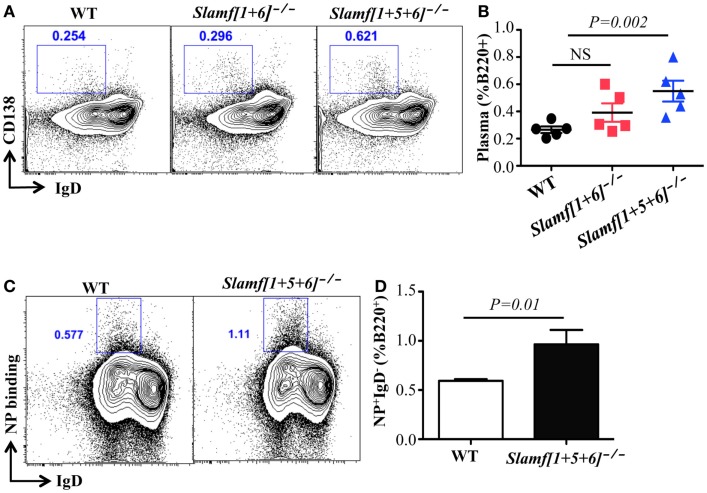
**A combined absence of SLAMF1, SLAMF5, and SLAMF6 enhances antigen specific plasma cell expansion**. *Slamf[1* + *6]*^−/−^*, Slamf[1* + *5* + *6]*^−/−^, and WT mice were immunized with NP-OVA in CFA. After 9 days, spleens were isolated and subjected to staining with the indicated antibodies and analyzed by flow cytometry. **(A)** Representative FACS plots showing B220^+^IgD^-^CD138^+^ plasma cells from the spleens of *Slamf[1* + *6]*^−/−^, *Slamf[1* + *5* + *6]*^−/−^, and WT mice. **(B)** Percentage of Plasma cells (B220^+^IgD^-^CD138^+^) from the spleens of *Slamf[1* + *6]*^−/−^, *Slamf[1* + *5* + *6]*^−/−^, and WT mice. **(C)** Representative FACS plots showing B220^+^NP^+^IgD^-^ NP-specific plasma cells from the spleens of *Slamf[1* + *5* + *6]*^−/−^ and WT mice. **(D)** Percentage of NP-staining plasma cells (B220^+^NP^+^IgD^-^) from the spleens of *Slamf[1* + *5* + *6]*^−/−^ and WT mice. Data represent at least three independent experiments.

Given the increased antibody responses observed in *Slamf[1* + *5* + *6]*^−/−^ mice, we hypothesized that the absence of SLAMF, SLAMF5, and SLAMF6 may enhance either plasma cell differentiation or their capacity to produce antibody. To test this hypothesis, we evaluated NP-specific plasma cells in NP-OVA immunized mice. Consistent with high antibody responses, the frequency of plasma cells (B220^+^IgD^-^CD138^+^) was significantly increased in *Slamf[1* + *5* + *6]*^−/−^ mice (Figures 4A,B). Furthermore, flow cytometric analysis confirmed an increase in NP-specific plasma cells in immunized *Slamf[1* + *5* + *6]*^−/−^ mice as compared to wild-type mice (Figures 4C,D). Together, the data indicate that the absence of SLAMF1, SLAMF5, and SLAMF6 has no effect on Tfh and GC B cell development, but that it appears to regulate development of antigen specific plasma cells.

A new Treg cell subset termed Tfr cells has recently been identified (49, 50). These cells, which express not only CXCR5 and PD-1 but also the transcription factors Bcl6 and FoxP3, suppress both Tfh cells and GC B cells. As Tfr cell differentiation requires SAP expression (49), it is possible that the absence of SLAMF1, SLAMF5, and SLAMF6 might also cause a defect in Tfr cell development, thereby contributing to the enhanced antibody responses in *Slamf[1* + *5* + *6]*^−/−^ mice. To assess the impact of the combined absence of SLAMF1, SLAMF5, and SLAMF6 on Tfr cell differentiation, we immunized mice with NP-OVA and analyzed Tfr cells 7 days later. The frequency of Tfr cells (CD4^+^CXCR5^high^PD-1^high^FoxP3^+^) was not significantly affected in *Slamf[1* + *6]*^−/−^ or *Slamf[1* + *5* + *6]*^−/−^ mice (Figure S3A in Supplementary Material). Although the expression of Ki67, a marker used to identify proliferating cells, is slightly decreased in *Slamf[1* + *5* + *6]*^−/−^ Tfr cells, it is not statistically significant (Figure S3B in Supplementary Material). Thus, while the enhanced antibody production may not result from a defect in Tfr differentiation, its functional inability would not be excluded in *Slamf[1* + *5* + *6]*^−/−^ mice.

### *Slamf[1* + *5* + *6]*^−/−^ B or T cells adoptively transferred to *RAG-1* deficient mice can induce enhanced antibody responses

**Figure 5:**
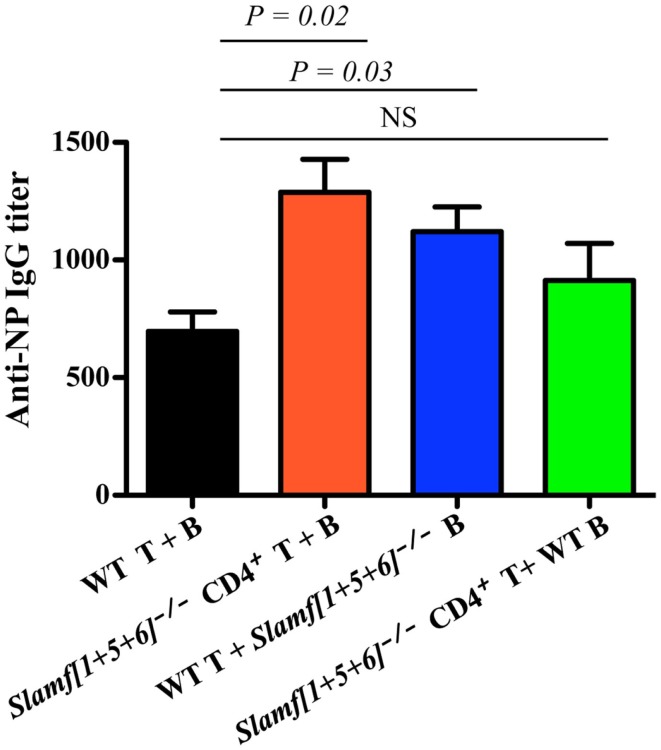
**The adoptive transfer of naïve *Slamf[1* + *5* + *6]^−/−^* T or B cells enhanced NP-specific antibody responses after co-transfer of WT B or T cells into *Rag-1^−/−^* mice**. CD4^+^ T cells (5 × 10^6^) together with 10 × 10^6^ B cells are isolated from WT and *Slamf[1* + *5* + *6]^−/−^* mice and transferred into *Rag-1^−/−^* recipients in the following four combinations of T and B cells: WT CD4^+^ T and WT B cells, *Slamf[1* + *5* + *6]^−/−^* CD4^+^ T and *Slamf[1* + *5* + *6]^−/−^* B cells, WT CD4^+^ T and *Slamf[1* + *5* + *6]^−/−^* B cells, and *Slamf[1* + *5* + *6]^−/−^* CD4^+^ T and WT B cells. The *Rag-1^−/−^* recipients were immunized with 40 μg of NP-OVA in CFA 7 days after the transfer. Mice were sacrificed and NP-specific IgG titers were determined by ELISA day 9 post-immunization. Data are representative of three independent experiments.

As SLAMF1, SLAMF5, and SLAMF6 are expressed on both B cells and T cells, it was not clear on which cell type ablation of their expression was critical for the altered T-dependent antibody responses observed in *Slamf[1* + *5* + *6]*^−/−^ mice. This prompted us to evaluate potential contributions of *Slamf[1* + *5* + *6]*^−/−^ T and B cells to the enhanced humoral responses by using the adoptive transfer of naïve T and B cells. To this end, four combinations of CD4^+^ cells and B cells were transferred into *Rag-1*^−/−^ recipient mice: WT CD4^+^ T and WT B cells, *Slamf[1* + *5* + *6]*^−/−^ CD4^+^ T and *Slamf[1* + *5* + *6]*^−/−^ B cells, WT CD4^+^ T and *Slamf[1* + *5* + *6]*^−/−^ B cells, and *Slamf[1* + *5* + *6]*^−/−^ CD4^+^ T and WT B cells. Seven days post-transfer, the recipient *Rag-1^−/−^* mice were immunized with NP-OVA in CFA. *Rag-1^−/−^* mice reconstituted with CD4^+^ T cells and B cells from *Slamf[1* + *5* + *6]^−/−^* mice had significantly higher NP-specific antibody production than recipient mice that had been reconstituted with WT CD4^+^ T cells and B cells (Figure 5). Interestingly, the transfer of *Slamf[1* + *5* + *6]^−/−^* B cells together with WT CD4^+^ T cells was sufficient to induce a stronger antibody response as compared to the transfer of WT CD4^+^ T cells and B cells. Although the transfer of *Slamf[1* + *5* + *6]^−/−^* CD4^+^ T cells and WT B cells also led to increased titers of NP-specific IgG, the magnitude of the antibody responses was less pronounced (Figure 5). Thus, we concluded that *Slamf[1* + *5* + *6]^−/−^* T and B cells both have intrinsic activity, but that the enhanced T-dependent humoral response in *Slamf[1* + *5* + *6]^−/−^* mice mainly results from *Slamf[1* + *5* + *6]^−/−^* B cells.

### Enhanced T-independent antibody responses are observed in *Slamf[1* + *5* + *6]^−/−^* mice

**Figure 6:**
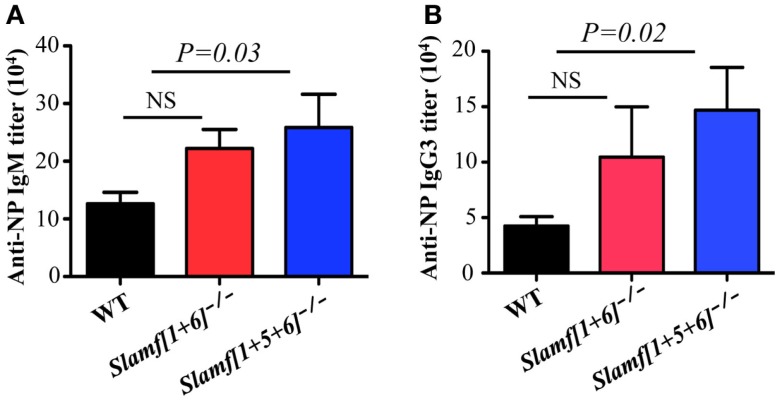
**A combined ablation of SLAMF1, SLAMF5, and SLAMF6 shows a selective increase in MZ B cells and enhanced TI-2 antibody responses**. WT, *Slamf[1* + *6]^−/−^*, and *Slamf[1* + *5* + *6]^−/−^* mice were immunized with 20 μg of NP-Ficoll. NP-specific IgM **(A)** and IgG3 **(B)** titers were determined at day 7 by ELISA after serial dilutions of the serum. Results are representative of three independent experiments.

Because *Slamf[1* + *5* + *6]^−/−^* mice have a high frequency of MZ B cells (Figures 2A,B) that are known to participate in responses to T-independent antigens, we questioned whether T-independent antibody responses are affected in *Slamf[1* + *5* + *6]^−/−^* mice. Consequently, we examined the SLAMF mutant mice in response to NP-Ficoll, a classical synthetic TI-2 antigen that induces murine Ag-specific B cells to expand, differentiate, and produce NP-specific antibodies. As anticipated, the serum concentrations of NP-specific IgM and IgG3 in *Slamf[1* + *5* + *6]^−/−^* mice were increased on day 7 after immunization compared to those in WT mice (Figures 6A,B). In contrast, NP-specific IgM and IgG3 titers were comparable in WT and *Slamf[1* + *6]^−/−^* mice (Figures 6A,B). As responses to NP-LPS were not altered (data not shown), the enhanced TI-2 responses are specifically linked to the combined absence of SLAMF1, SLAMF5, and SLAMF6.

### Anti-SLAMF6 antibody inhibits humoral immune responses

**Figure 7:**
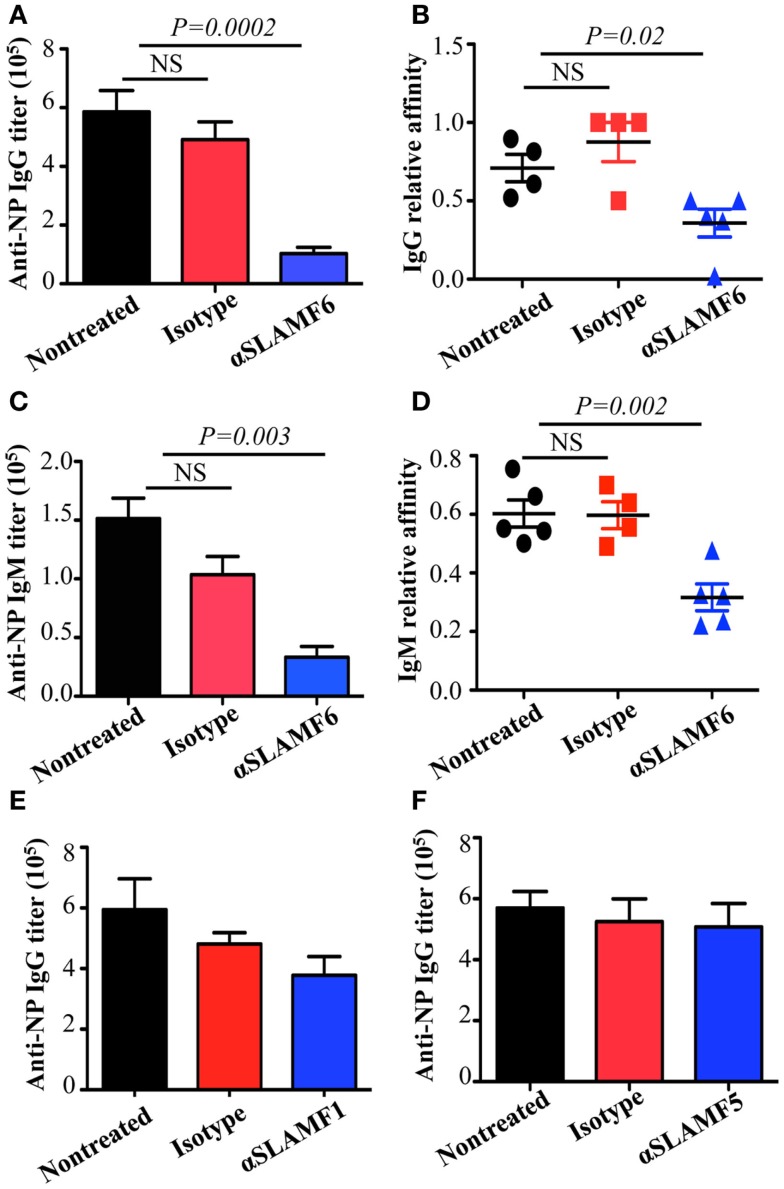
**Administration of anti-SLAMF6 antibody has a negative effect on antibody production in protein-immunized WT mice**. Mice were immunized with 40 μg NP-OVA in CFA and some were injected with either 250 μg of anti-SLAMF6 (330), anti-SLAMF1 (9D1), anti-SLAMF5 (M5), or mouse Ig isotype control. The mice were sacrificed on day 9 and serum was collected to measure Ig production. **(A)** NP-specific IgG titers from sera of anti-SLAMF6 injected mice were determined by ELISA. **(B)** Affinity of NP-specific IgG in immune-sera collected as in **(A)**. **(C)** NP-specific IgM titers from sera of anti-SLAMF6 injected mice were determined by ELISA. **(D)** Affinity of NP-specific IgM in immune-sera collected as in **(C)**. **(E)** NP-specific IgG titers from sera of anti-SLAMF1 injected mice were determined by ELISA. **(F)** NP-specific IgG titers from sera of anti-SLAMF5 injected or non-injected immunized mice were determined by ELISA. Results are representative of three independent experiments.

**Figure 8:**
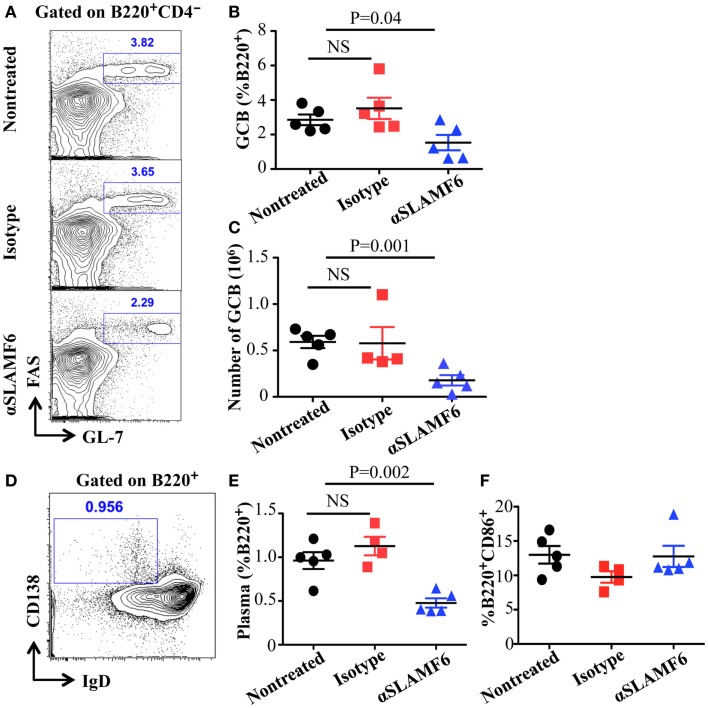
**Administration of anti-SLAMF6 (330) antibody has a negative effect on GC B cell differentiation in protein-immunized WT mice**. Mice were immunized with 40 μg of NP-OVA in CFA and some mice were injected with either 250 μg anti-SLAMF6 (330) or Ig isotype control. The mice were sacrificed on day 9. **(A)** Representative flow cytometry staining of B220^+^GL7^+^Fas^+^ Germinal Center B cells in the spleens of anti-SLAMF6, isotype, and non-injected immunized mice. **(B)** Percentage of Germinal Center B cells (B220^+^GL7^+^Fas^+^) in the spleens of anti-SLAMF6, isotype, and non-injected immunized mice was determined by flow cytometry. **(C)** The numbers of Germinal Center B cells (B220^+^GL7^+^Fas^+^) in the spleens of anti-SLAMF6, isotype, and non-injected immunized mice were determined by flow cytometry. **(D)** Representative of flow cytometry staining of B220^+^IgD^-^CD138^+^ plasma cells in the spleens of anti-SLAMF6, isotype, and non-injected immunized mice. **(E)** Percentage of plasma cells (B220^+^IgD^-^CD138^+^) in the spleens of anti-SLAMF6, isotype, and non-injected immunized mice. **(F)** Percentage of B220^+^CD86^+^ activated B cells in the spleens of anti-SLAMF6, isotype, and non-injected immunized mice. Results are representative of three independent experiments.

**Figure 9:**
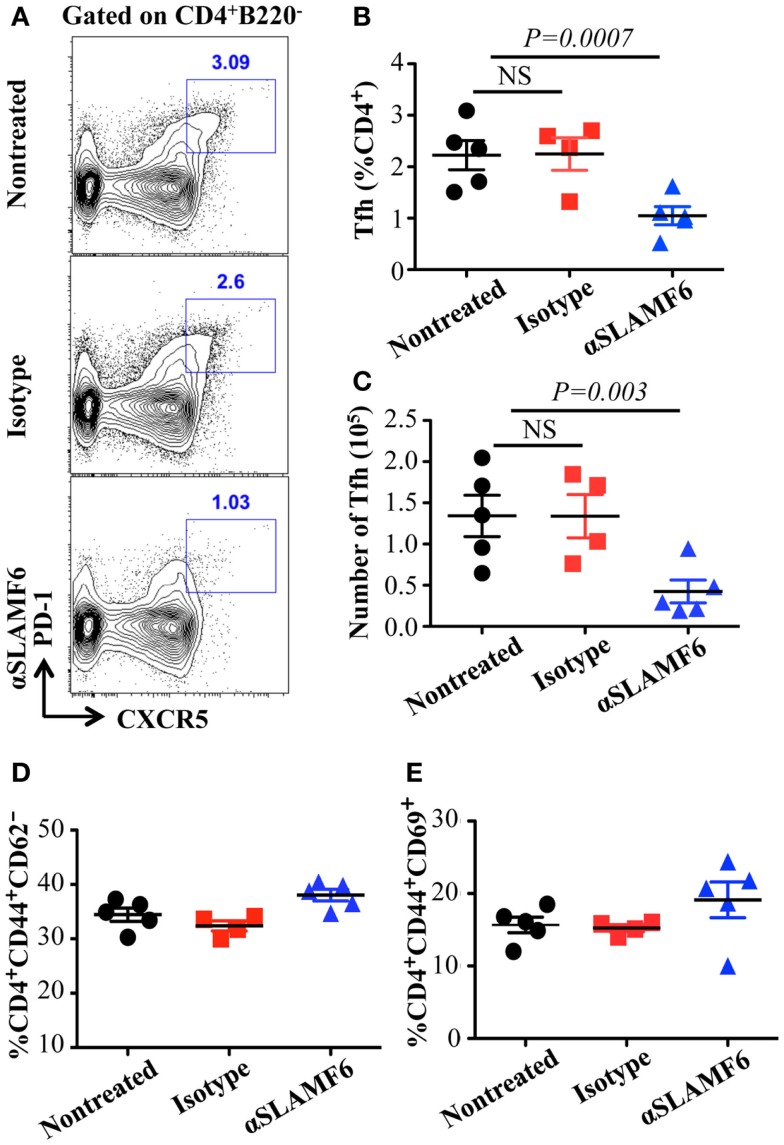
**Administration of anti-SLAMF6 (330) antibody has a negative effect on Tfh cell differentiation in protein-immunized WT mice**. Mice were immunized with 40 μg of NP-OVA in CFA and some mice were injected with either 250 μg anti-SLAMF6 (330) or Ig isotype control. The mice were sacrificed on day 9. **(A)** Representative flow cytometry staining of CD4^+^PD-1^+^CXCR5^+^ Tfh cells in the spleens of anti-SLAMF6, isotype, and non-injected immunized mice. **(B)** Percentage of Tfh cells (CD4^+^PD-1^+^CXCR5^+^) was determined by flow cytometry in the spleens of anti-SLAMF6, isotype, and non-injected immunized mice. **(C)** The number of Tfh cells (CD4^+^PD-1^+^CXCR5^+^) was determined by flow cytometry in the spleens of anti-SLAMF6, isotype, and non-injected immunized mice. **(D)** Percentage of CD4^+^CD44^hi^CD62^lo^ memory T cells in the spleens of anti-SLAMF6, isotype, and non-injected immunized mice. **(E)** Percentage of CD4^+^CD44^hi^CD69^hi^ activated T cells in the spleens of anti-SLAMF6, isotype, and non-injected immunized mice. Results are representative of three independent experiments.

The observation that the ablation of SLAMF1, SLAMF5, and SLAMF6 enhances T-dependent and T-independent antibody responses suggests that they function as potential inhibitory molecules in humoral immune responses. In addition, a recent report indicates that SLAMF6 transmits inhibitory signaling in Tfh differentiation and NKT development in the context of the absence of SAP (27). To directly evaluate whether triggering of a single SLAMF receptor would initiate inhibitory signaling in humoral responses, we first sought to trigger SLAMF6 by using anti-SLAMF6 mAb in NP-OVA-immunized mice. As shown in Figure 7, the treatment of WT mice with anti-SLAMF6 (330) dramatically impaired NP-specific IgG production 9 days post-immunization of NP-OVA (Figure 7A). As negative control, anti-SLAMF6 had no detectable effects on antibody production in *Slamf[1* + *6]^−/−^* mice (Figure S4A in Supplementary Material). Noticeably, anti-SLAMF6 injected mice had a significant reduction in IgG high affinity antibody (Figure 7B). Furthermore, NP-specific IgM production and its affinity maturation also were impaired in anti-SLAMF6 injected mice (Figures 7C,D). In correlation with the impaired antibody response, the frequencies and absolute numbers of splenic GC B cells (GL7^+^Fas^+^) (Figures 8A–C) and Tfh T cells (CXCR5^+^PD-1^+^) (Figures 9A–C) were significantly reduced in anti-SLAMF6 injected mice. A reduced number of plasma cells was also observed in anti-SLAMF6 injected mice (Figures 8D,E), but effector B cell and T cell populations were equivalent in anti-SLAMF6 injected and non-injected mice (Figure 8F and Figures 9D,E). In order to confirm the inhibitory effects of anti-SLAMF6 (330), another anti-SLAMF6 mAb (13G3) was also tested. As expected, a similar inhibitory effect was seen in 13G3-injected mice (Figures S4A,B in Supplementary Material).

To address whether the administration of anti-SLAMF6 affects early commitment to antigen specific Tfh cells and GC B cells or late stages of humoral responses, immunized mice were injected with anti-SLAMF6 four days post-immunization, at which point T and B cells are already committed to becoming Tfh and GC B cells (51). Interestingly, the late injection of mice with anti-SLAMF6 did not significantly reduce GC response and antibody production (data not shown). This suggests that the signal initiated by SLAMF6 has efficient inhibition in early Tfh and GC B cell differentiations, but has little effect on late Tfh and GC B cell expansion and antibody production.

**Figure 10:**
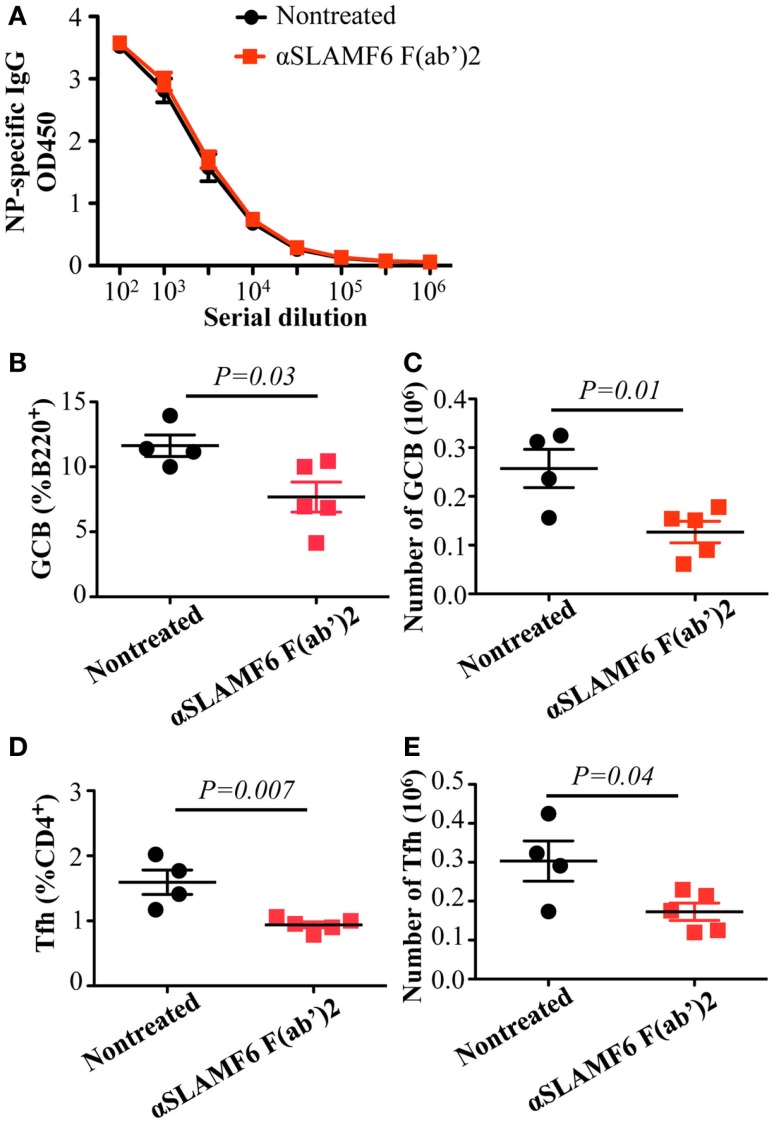
**Administration of anti-SLAMF6 (330) F(ab’)_2_ antibody has a similar negative effect on GC B cell and Tfh cell development in protein-immunized WT mice**. Mice were immunized with 40 μg of NP-OVA in CFA and some were injected with 250 μg of anti-SLAMF6 F(ab’)_2_ on day 0 and day 4. The mice were sacrificed on day 9 and serum was collected to measure IgG production. **(A)** NP-specific IgG titers were determined by ELISA. **(B–C)** The percentage and number of Germinal Center B cells (B220^+^GL7^+^Fas^+^) were determined by flow cytometry. **(D–E)** The percentage and number of Tfh cells (CD4^+^PD-1^+^CXCR5^+^) were determined by flow cytometry. Results are representative of three independent experiments.

To evaluate whether the Fc portion of anti-SLAMF6 influences immune function, anti-SLAMF6 F(ab’)_2_ fragments were injected into mice along with NP-OVA immunization. Similar to intact anti-SLAMF6, anti-SLAMF6 F(ab’)_2_ caused a significant decrease in the percentage and number of GC B cells (Figures 10B,C) and Tfh cells (Figures 10D,E). In contrast, an impaired NP-specific antibody production was not observed in the anti-SLAMF6 F(ab’)_2_ injected mice. To exclude the possibility that NK cells mediate natural cytotoxicity against a variety of immune cells, we examined splenocyte phenotype and levels of NP-specific antibody in NK-depleted WT mice following NP-OVA immunization and anti-SLAMF6 injection. Notably, the depletion of NK cells did not impact the capacity of anti-SLAMF6 to suppress antibody production or the development of Tfh cells and GC B cells (data not shown). Thus, severely impaired antibody production by the injection of anti-SLAMF6 is not due to NK cell-mediated ADCC.

Based on the studies using anti-SLAMF6, we next examined the ability of anti-SLAMF1 mAb and anti-SLAMF5 mAb to suppress humoral immune responses in NP-OVA immunized mice. We found that NP-specific antibody production was moderately, but consistently, reduced in anti-SLAMF1 injected mice (Figure 7E). However, anti-SLAMF5 did not suppress antibody production (Figure 7F). In addition, the frequencies of GC B cells and Tfh cells were comparable among the non-injected, anti-SLAMF1, and anti-SLAMF5 injected mice (data not shown). Thus, the results suggest that neither anti-SLAMF1 nor anti-SLAMF5 is alone sufficient to have a significant impact on humoral responses.

## Discussion

An important role of SAP in T cell help to B cells has been highlighted in defects in follicular T helper cell differentiation and lack of germinal center development in XLP patients and in virally infected or immunized *SAP^−/−^* mice (4, 12, 13, 32, 48, 52). Although SAP has been proven to bind to ITSMs in most SLAMF receptors upon ligand stimulation, how these receptors function in the presence and absence of SAP is poorly understood, particularly in B cells. In this study, we provide *in vivo* evidence that SLAMF1, SLAMF5, and SLAMF6 synergistically suppress T-dependent and T-independent antibody responses, as *Slamf[1* + *5* + *6]^−/−^* mice, but not *Slamf[1* + *6]^−/−^* mice or single SLAMF mutant mice, exhibit a significant enhancement of antibody production. Strikingly, an adoptive transfer assay shows that the enhanced antibody responses depend largely on *Slamf[1* + *5* + *6]^−/−^* B cells, which indicates that the absence of SLAMF1, SLAMF5, and SLAMF6 induces intrinsic B cell activity. Furthermore, injection of mice with anti-SLAMF6 mAb dramatically reduced antibody responses accompanied by impairing Tfh cell and GC B cell development in spite of a less suppressive effect of anti-SLAMF1 mAb on antibody responses. Our results therefore point to a new and important mechanism by which SLAMF1, SLAMF5, and SLAMF6 regulate humoral responses in B cells.

Maintaining sustained CD4^+^ T cell adhesion to B cells is required for Tfh differentiation and germinal center development, which allows for important signal transfer between T and B cells. SAP-deficient T cells fail to form a stable T-B cell conjugate (41) and thereby severely impaired development of Tfh cells and GC B cells becomes a hallmark in XLP patients and *SAP^−/−^* mice (14, 32, 34, 41). Although most SAP-binding SLAMF receptors are highly expressed on resting and activated T and B cells and implicate a diverse array of lymphocyte functions, including sustained T-B cell conjugates (43, 53), the deficiencies in single SLAMF receptors actually exhibit mild phenotypes. However, in contrast to our current study, *Slamf5^−/−^* mice have previously been shown to have a defect in germinal center development and T-dependent antibody production in protein immunization (43). The reason that the same B6 background *Slamf5^−/−^* mice strains have different phenotypes is not clear. One explanation for this discrepancy is that the presence of selection marker (Neo) could influence neighboring genes in targeted loci (54). Alternatively, it remains possible that the discordant findings could reflect the different environmental conditions of animal facilities.

Because the functional redundancies in SLAMF-mediated signaling have been demonstrated in NKT cell development (45, 46), we speculate that multiple deficiencies in SLAMF receptors may have a strong influence on humoral immune responses that are able to recapitulate most of the phenotypic alterations observed in *SAP^−/−^* mice. Surprisingly, in our *in vivo* studies comparing T-dependent antibody responses, loss of expression of SLAMF1, SLAMF5, and SLAMF6 receptors actually removed inhibitory signaling and resulted in higher antibody responses (Figure 3A). When the antibody responses in mice lacking *Slamf[1* + *6]* were compared to those in WT mice, there was also a consistent increase in NP-specific antibody titer, but the effect was less pronounced than that observed in the combined ablation of SLAMF1, SLAMF5, and SLAMF6. Although some variability in antibody responses between mutant and WT mice occurs, statistical analysis always reached significance. These findings indicate that the homophilic binding of SLAMF1, SLAMF5, and SLAMF6 synergistically transmits inhibitory signaling during humoral immune responses. The dual function of SLAMF receptors was initially reported in NK cell studies. In human NK cells, SLAMF4 predominantly functions as an activating receptor because engagement of SLAMF4 with SLAMF2 mediates NK cell cytotoxicity, cell proliferation, and cytokine secretion. However, in NK cells from XLP patients, the SLAMF2–SLAMF4 interaction fails to activate NK cells, but rather inhibits NK-medicated cytolysis (23). Besides SLAMF4, other SLAMF receptors such as SLAMF3, SLAMF5, and SLAMF6 become inhibitory molecules instead of activating receptors in mouse NK cells lacking SAP, EAT-2A, and EAT-2B (55). Consistent with these findings, a similar inhibitory effect of SLAMF6 on humoral responses and NKT cell development was reported in the context of the absence of SAP (27). Collectively, these observations suggest that most SLAMF receptors can mediate either positive or negative signaling, depending on the expression of SLAMF adaptors, SAP and EAT-2. Given that SAP and EAT-2A/B are not expressed in B cells, the homophilic interactions of SLAMF1, SLAMF5, and SLAMF6 between T and B cells or B and B cells would result in preferential binding to inhibitory signaling molecules (e.g., SHP1) in B cells because of the lack of competition of SAP and EAT-2 for ITSMs of SLAMF receptors. This idea is supported by our adoptive transfer assays, in which B cells from *Slamf[1* + *5* + *6]^−/−^* mice led to a further enhancement in antibody responses compared to the transfer of *Slamf[1* + *5* + *6]^−/−^* T cells. Furthermore, *Slamf[1* + *5* + *6]^−/−^* mice display higher antibody responses in the absence of T cell help when immunized with T-independent antigen NP-Ficoll, which directly indicates that an intrinsic B cell hyperactivation exists in *Slamf[1* + *5* + *6]^−/−^* mice.

Although the higher antigen specific antibody production does not accompany enhanced Tfh and GC B cell responses in the absence of SLAMF1, SLAMF5, and SLAMF6, increased development of plasma cells is consistently observed in NP-OVA immunized *Slamf[1* + *5* + *6]^−/−^* mice (Figures 4A–D). The mechanism regulating plasma cell differentiation is only partly understood. Two transcription factors Bcl-6 and Blimp-1 reciprocally modulate differentiation of GC B cells and plasma cells (52). Cytokines and chemokines also provide crucial survival signals to plasma cells (56). Far less is known about SLAMF receptor-mediated signals for plasma cell differentiation and function. However, in our experiments, we provide interesting evidence that SLAMF1, SLAMF5, and SLAMF6 negatively regulate either plasma cell differentiation and/or expansion in humoral immune responses. Further work will assess how SLAMF receptors are involved in plasma cell development at a molecular level.

The interesting finding in this study is that *Slamf[1* + *5* + *6]^−/−^* mice exhibit an increased frequency of MZ B cells. A cell-fate decision between follicular B cells and MZ B cells occurs in the transitional (T2) B cell stage, when T2 B cells differentiate into follicular B cells or MZ B cells after integration of BCR signal strength and signaling via other essential signaling molecules (57–59). Like in GC B cells, SLAMF1, SLAMF5, and SLAMF6 are highly expressed in transitional B cells and MZ B cells (ImmGen.org), and therefore, signaling resulting from their homophilic interaction may implicate differentiation, migration, or survival of MZ B cells. Since differentiation of transitional B cells was not altered in *Slamf[1* + *5* + *6]^−/−^* mice, signaling initiated by SLAMF1, SLAMF5, and SLAMF6 seems to play a critical role in controlling the development and/or survival of MZ B cells. It has been described that MZ B cells and B1 cells are prime B cell subpopulations responding to T-independent antigens (60, 61). Interestingly, in spite of increased pools of MZ B cells, *Slamf[1* + *5* + *6]^−/−^* mice showed enhanced immune responses to the TI-2 Ag NP-Ficoll, but not to the TI-1 Ag NP-LPS. The fact that enhanced TI-antigen response is only limited to TI-2 antigens suggests that *Slamf[1* + *5* + *6]^−/−^* MZ B cell intrinsic activity, not number, seems to be more critical in determining the extent of humoral immunity. Thus, these results provide evidence that synergistic activity of SLAMF1, SLAMF5, and SLAMF6 may be implicated in functional activity of MZ B cells.

Complementary approaches with SLAMF receptor-deficient mice and SLAMF-specific antibodies are important for understanding the functions of their immunoregulatory pathways. Recently, SLAMF6 was found not only to constitutively associate with SHP1 in SAP-sufficient cells, but also to co-distribute with the CD3 complex. The ligation of SLAMF6 can reduce CD3ζ phosphorylation (53). Based on these findings, we hypothesize that crosslinking of SLAMF6 by anti-SLAMF6 mAb may cause high phosphorylation in its ITSM. Subsequently, protein tyrosine phosphatases and lipid phosphatases are preferentially recruited to SLAMF6, particularly in SAP and EAT-2 negative B cells. Surprisingly, we found that treatment of WT mice with anti-SLAMF6 almost recapitulates the phenotype observed in *SAP^−/−^* mice, which showed a marked defect in Tfh cell and GC B cell formation and reduced antibody production and affinity maturation. Interestingly, a significant defect in humoral response was not observed when mice were treated with anti-SLAMF6 four days after antigen exposure. This further indicates that SLAMF6-mediated inhibitory signals have distinct roles in the early differentiation of Tfh cells and GC B cells. However, the administration of anti-SLAMF6 F(ab’)_2_ fragments did not fully suppress NP-specific antibody production even though the development of Tfh cells and GC B cells was significantly impaired (Figure 10). This difference may be due to a shorter half-life of anti-SLAMF6 F(ab’)_2_, which could prevent it from maintaining sustainable triggering of SLAMF6 receptors during *in vivo* immune responses. Alternatively, the suppressive effect of anti-SLAMF6 mAb on humoral response might depend on the ability of anti-SLAMF6 Fc to bind to other accessory cells and crosslink the SLAMF6 receptors on Tfh cells and GC B cells. Such crosslinking is necessary for many surface molecules to initiate signaling events. Clearly, further studies are required to determine the contribution of other mechanisms such as complement and non-NK cell-mediated cytotoxicity in suppressing the activity of anti-SLAMF6 mAb. Compared to anti-SLAMF6 mAb, anti-SLAMF1 mAb has a milder, yet consistent, negative effect on antibody production, but anti-SLAMF5 mAb does not show any impact on humoral response. It seems contradictory to the functional redundancy we observed in *Slamf[1* + *5* + *6]^−/−^* mice. In fact, SLAMF6 has been shown to initiate dominant signaling in SAP*^−/−^* mice because ablation of the *Slamf6* gene, but not the *Slamf1* gene or *Slamf5* gene, can rescue Tfh cell differentiation and antibody responses in *SAP^−/−^* mice (27, 44). This indicates that SLAMF6 can complement deficiency of either SLAMF1 or SLAMF5 to facilitate inhibitory signaling in the absence of SAP. However, if *Slamf1* and *Slamf5* double mutations can be introduced into *SAP^−/−^* mice, impaired humoral responses may be partially restored. Similarly, the injection of mice with both anti-SLAMF1 mAb and anti-SLAMF5 mAb may also cause some reduction in antibody production.

**Figure 11:**
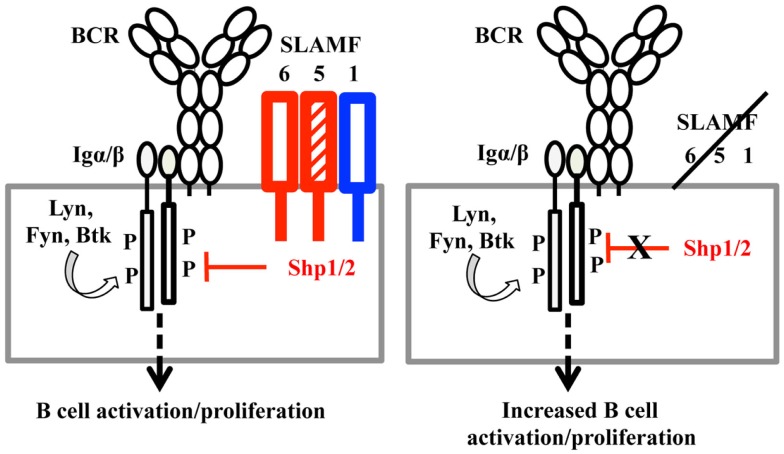
**A model of SLAMF receptors: SHP1/2 action on the BCR during B cell activation**. When B cells are activated, the ITSMs of SLAMF1, SLAMF5, and SLAMF6 recruit SHP1/2 and translocate these phosphatases to the vicinity of the B cell antigen receptor. Signaling from the BCR is thus down-regulated, maintaining proper response to antigens in humoral responses. When SLAMF1, SLAMF5, and SLAMF6 are deleted from B cells, inhibitory signaling mediated by SLAMF and SHP1/2 is dampened, which induces enhanced humoral responses.

By comparing T-dependent and T-independent antigen responses in *Slamf5^−/−^*, *Slamf[1* + *6]^−/−^*, and *Slamf[1* + *5* + *6]^−/−^* mice, we demonstrated for first time that SLAMF1, SLAMF5, and SLAMF6 synergistically regulate humoral immune responses. Genetic interruption of SLAMF1, SLAMF5, and SLAMF6 results in enhanced antibody responses to T-dependent and T-independent antigens. In complementary studies, the administration of anti-SLAMF6 mAb further implicates SLAMF6 as a primary inhibitory member of the SLAMF receptors in antibody responses. The studies suggest that the ligation of SLAMF receptors in SAP-negative B cells (Figure 11) may preferentially recruit inhibitory signaling molecules to immunological synapses and control B cell responses during cognate interaction between T and B cells.

## Conflict of Interest Statement

The authors declare that the research was conducted in the absence of any commercial or financial relationships that could be construed as a potential conflict of interest.

## Supplementary Material

The Supplementary Material for this article can be found online at http://journal.frontiersin.org/article/10.3389/fimmu.2015.00158
