# Supplementary figures and images for: Negative Regulation of Humoral Immunity Due to Interplay between the SLAMF1, SLAMF5, and SLAMF6 Receptors
Source: Front Immunol. 2015 Apr 14;6:158. doi: 10.3389/fimmu.2015.00158 (PMC4396446; doi:10.3389/fimmu.2015.00158)

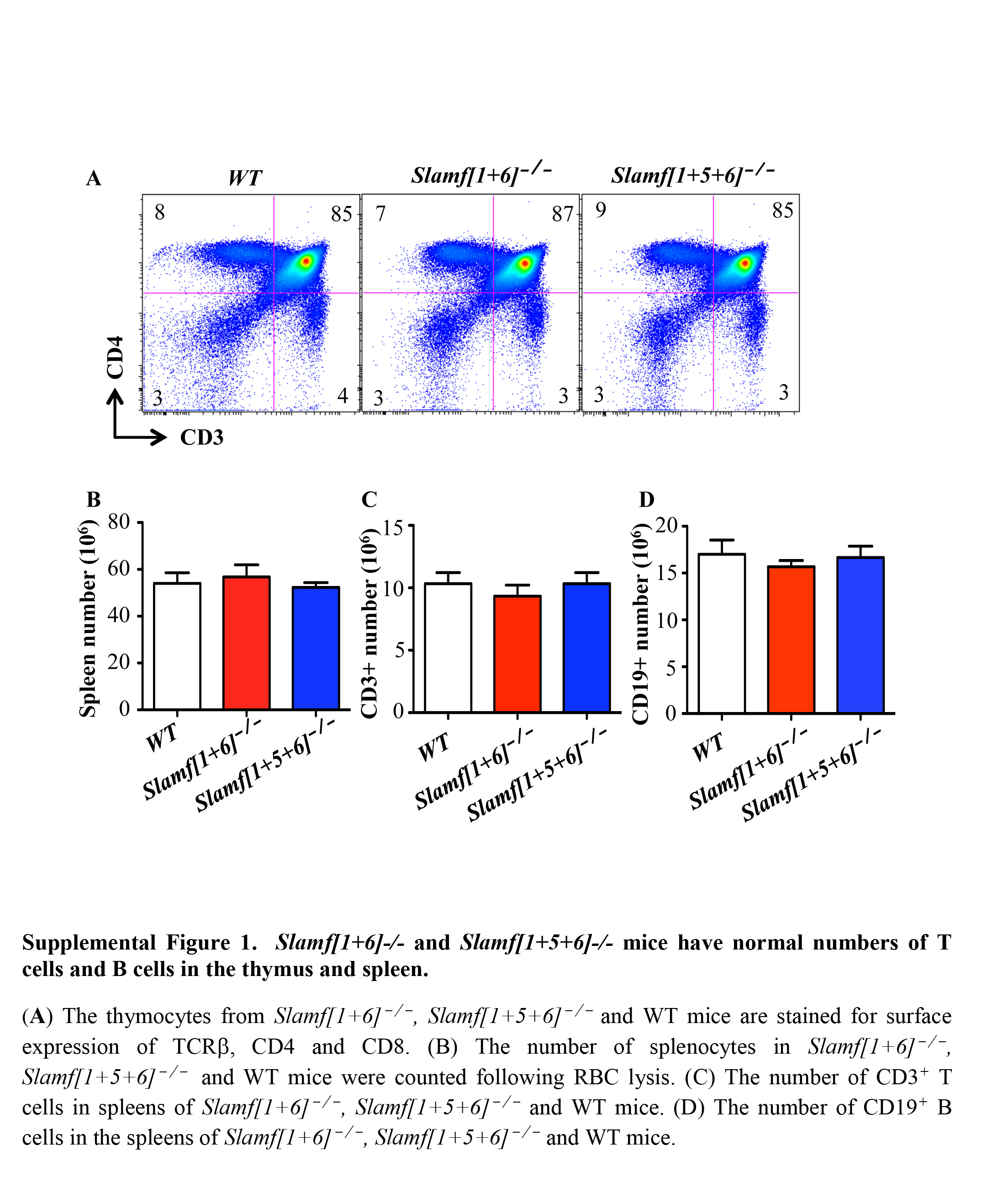

Supplement: Supplementary file 1 [file Image_1.JPEG]

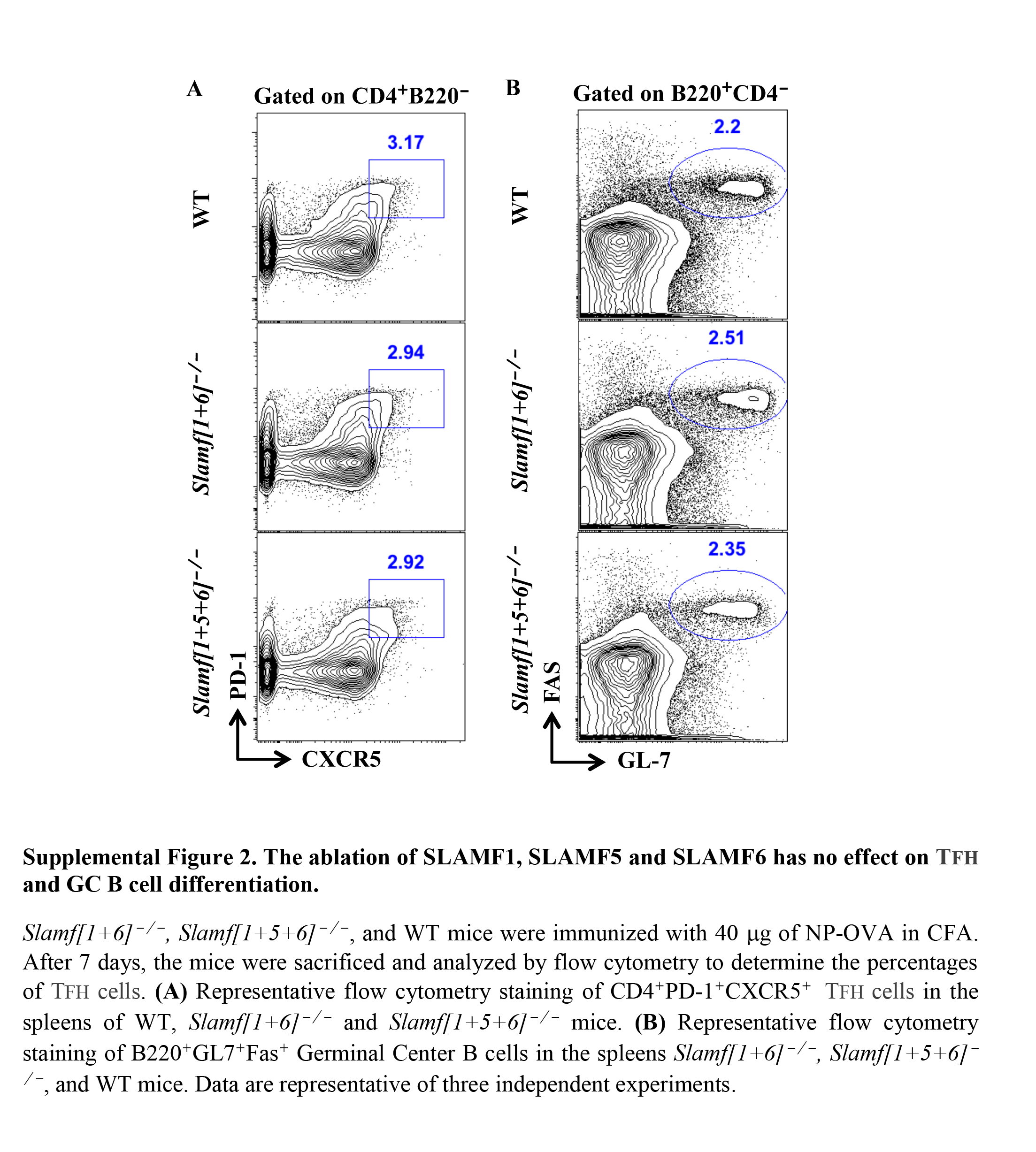

Supplement: Supplementary file 2 [file Image_2.JPEG]

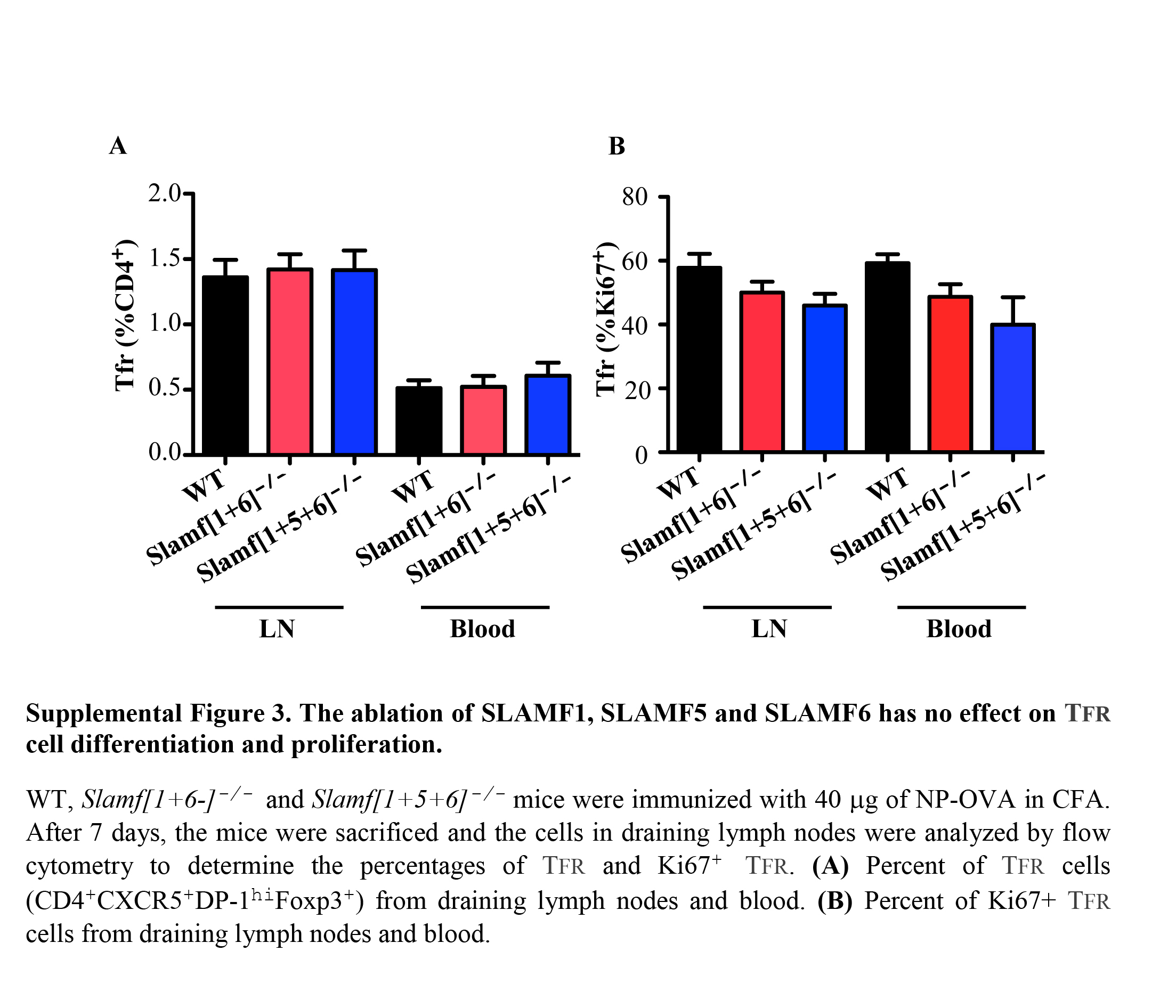

Supplement: Supplementary file 3 [file Image_3.JPEG]

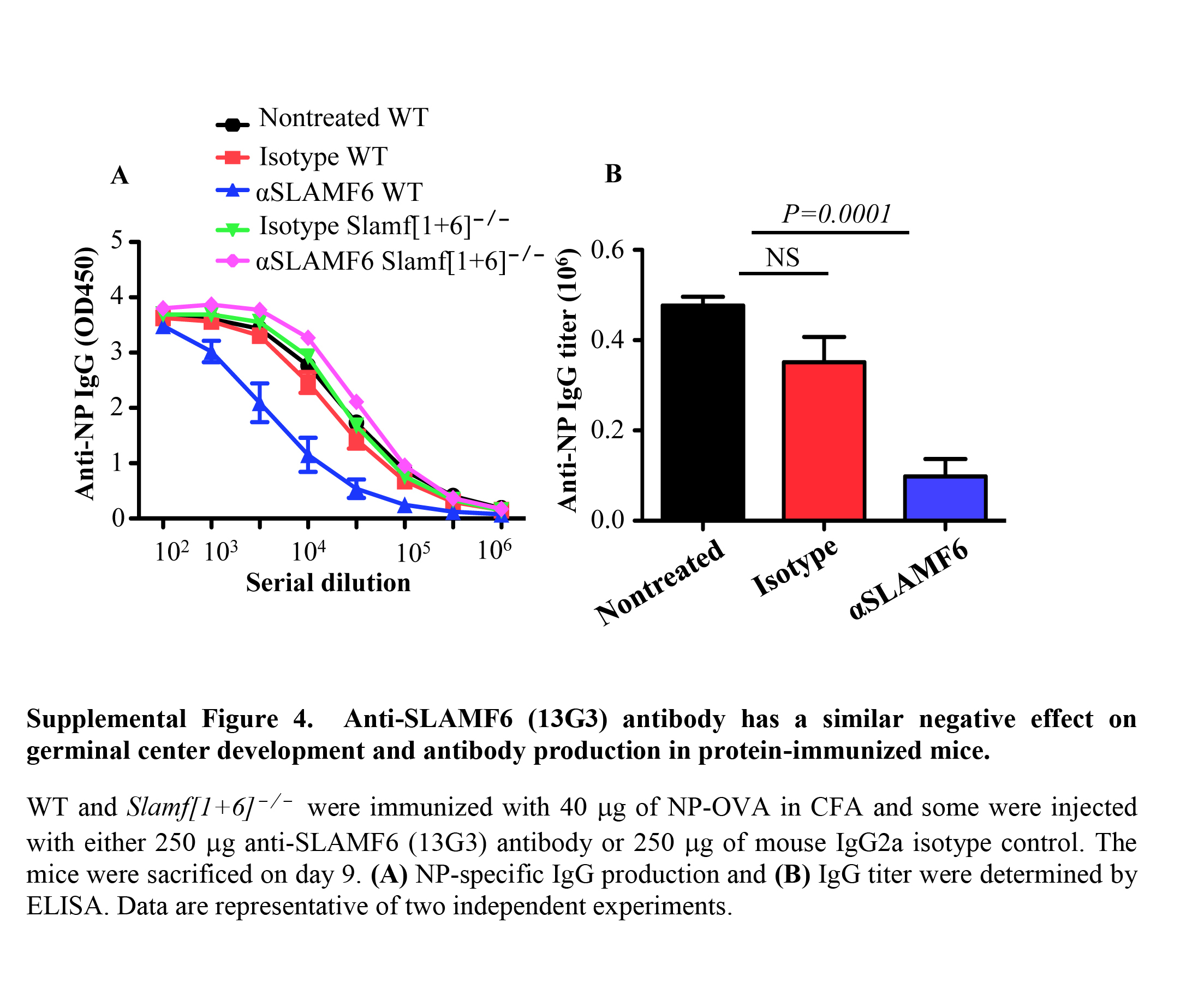

Supplement: Supplementary file 4 [file Image_4.JPEG]
